# Crystal structure and Hirshfeld surface analysis of 4-azido-2-(3,5-di­methyl­phen­yl)-5-(4-nitro­phen­yl)-2*H*-1,2,3-triazole

**DOI:** 10.1107/S2056989023007855

**Published:** 2023-09-14

**Authors:** Abel Maharramov, Namiq Q. Shikhaliyev, Afaq Abdullayeva, Gulnar T. Atakishiyeva, Ayten Niyazova, Victor N. Khrustalev, Shahnaz I. Gahramanova, Zeliha Atioğlu, Mehmet Akkurt, Ajaya Bhattarai

**Affiliations:** a Organic Chemistry Department, Baku State University, Z. Khalilov str. 23, AZ 1148 Baku, Azerbaijan; b Department of Engineering and Applied Sciences, Azerbaijan State University of Economics, M. Mukhtarov 194, Baku AZ1001, Azerbaijan; c Peoples’ Friendship University of Russia (RUDN University), Miklukho-Maklay St. 6, Moscow, 117198, Russian Federation; d N. D. Zelinsky Institute of Organic Chemistry RAS, Leninsky Prosp. 47, Moscow, 119991, Russian Federation; e Institute of Catalysis and Inorganic Chemistry , 113 H. Javid Ave., AZ1143 Baku, Azerbaijan; f Department of Aircraft Electrics and Electronics, School of Applied Sciences, Cappadocia University, Mustafapaşa, 50420 Ürgüp, Nevşehir, Türkiye; g Department of Physics, Faculty of Sciences, Erciyes University, 38039 Kayseri, Türkiye; h Department of Chemistry, M.M.A.M.C (Tribhuvan University) Biratnagar, Nepal; Venezuelan Institute of Scientific Research, Venezuela

**Keywords:** crystal structure, hydrogen bonds, azido group, 2*H*-1,2,3-triazole, Hirshfeld surface analysis

## Abstract

In the title compound, C_16_H_13_N_7_O_2_, the 3,5-di­methyl­phenyl and 4-nitro­phenyl rings are inclined to the central 2*H*-1,2,3-triazole ring by 1.80 (7) and 1.79 (7)°, respectively, and to one another by 2.16 (7)°. In the crystal, the mol­ecules are linked by C—H⋯N hydrogen bonds and π–π stacking inter­actions [centroid-to-centroid distances = 3.7295 (9) and 3.7971 (9) Å], forming ribbons along the *b*-axis direction. These ribbons are connected to each other by weak van der Waals inter­actions and the stability of the crystal structure is ensured. A Hirshfeld surface analysis of the crystal structure indicates that the most important contributions to the crystal packing are from H⋯H (31.5%), N⋯H/H⋯N (19.2%), O⋯H/H⋯O (14.5%), N⋯C/C⋯C (10.9%) and C⋯H/H⋯C (10.2%) contacts.

## Abstract 2

In the crystal, the mol­ecules are connected by C—H⋯N hydrogen bonds and π–π stacking inter­actions, forming ribbons along the *b*-axis direction. Weak van der Waals inter­actions link these ribbons together, consolidating the crystal structure.

## 1. Chemical context

**Figure 1:**
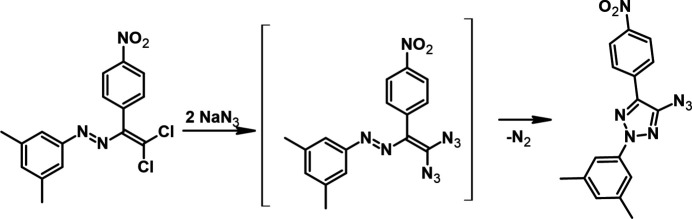
Reaction scheme for the synthesis of the title compound.

Triazoles are used as biological agents for their anti-inflammatory, anti-thrombotic and anti-viral activities (Blass *et al.*, 2006 ▸; Caliendo *et al.*, 1999 ▸; Phillips *et al.*, 2009 ▸). At the same time, 2*H*-1,2,3-triazoles are effective catalysts (Zhao *et al.*, 2008 ▸; Yan *et al.*, 2006 ▸; Chandrasekhar *et al.*, 2010 ▸) and are used as ionic liquids (Yoshida *et al.*, 2012 ▸) in organic synthesis. It should be noted that many methods for their preparation are quite complicated, which limits the possibilities of studying the biological activity of these compounds and also their use in other fields of science and technology. In general, the development of new synthesis methods for 2*H*-1,2,3-triazole derivatives has been a constant in the field of organic synthesis. Since the 1,2,3-triazole ring is an integral part of many medicinal preparations, research on their synthesis (Kamijo *et al.*, 2002 ▸; Liu *et al.*, 2008 ▸; Ghozlan *et al.*, 2006 ▸; Kalisiak *et al.*, 2008 ▸; Koszytkowska-Stawińska *et al.*, 2012 ▸) and biological activities is constantly increasing (Ferreira *et al.*, 2013 ▸; Tan *et al.*, 2002 ▸; Prusiner & Sundaralingam, 1973 ▸; Toniolo *et al.*, 2017 ▸; Caliendo *et al.*, 1999 ▸; Blass *et al.*, 2006 ▸; von Mutius *et al.*, 2012 ▸; Ferreira *et al.*, 2013 ▸). In particular, we can mention the synthesis of new 1,2,3-triazole-based drugs against tuberculosis (Sanna *et al.*, 2000 ▸). In this regard, the synthesis of 4-azido-2*H*-1,2,3-triazole derivatives (Tsyrenova *et al.*, 2021 ▸) from the reaction of di­chlorodi­aza­dienes with NaN_3_ is a relevant issue. In the following scheme (Fig. 1 ▸), on the basis of the synthesis of (*E*)-1-(2,2-di­chloro-1-(4-nitro­phen­yl)vin­yl)-2-(3,5-di­methyl­phen­yl) diazene obtained from the reaction of N-substituted hydrazone (Maharramov *et al.*, 2018 ▸; Nenajdenko *et al.*, 2020 ▸; Shikhaliyev *et al.*, 2018 ▸, 2019*a* ▸,*b* ▸, 2021*a* ▸,*b* ▸) and CCl_4_ with NaN_3_, 4-azido-2-(3,5-di­methyl­phen­yl)-5-(4-nitro­phen­yl)-2*H*-1,2,3-triazole was synthesized and its structure was confirmed by single-crystal X-ray analysis. 
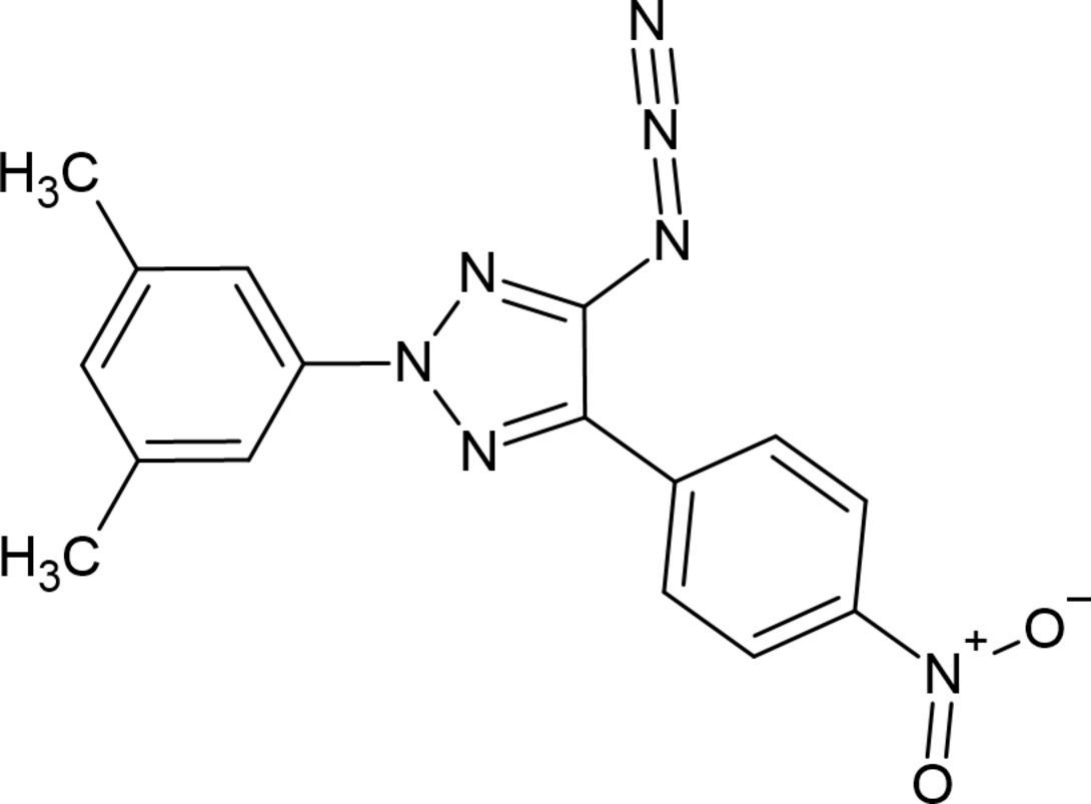


## 2. Structural commentary

**Figure 2:**
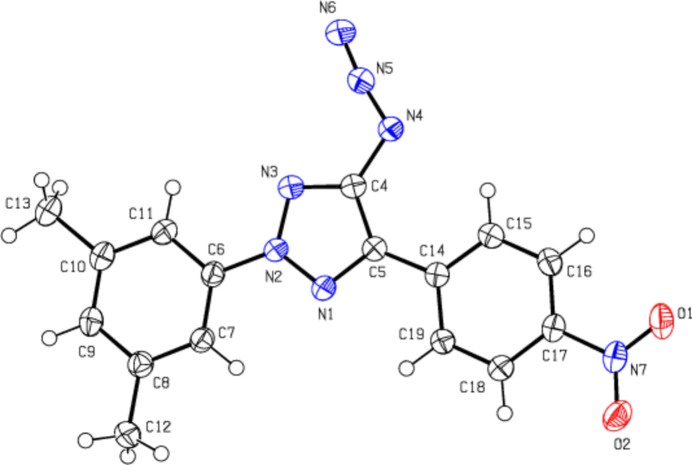
The mol­ecular structure of the title compound, showing the atom labelling and displacement ellipsoids drawn at the 50% probability level.

The mol­ecule of the title compound, (Fig. 2 ▸), except for the methyl H atoms, can be described as essentially planar [maximum deviations = 0.060 (1) Å for C15 and −0.076 (1) Å for N6] with the substituents rotated slightly around the triazole centre. The planar 3,5-di­methyl­phenyl (C6–C11) and 4-nitro­phenyl (C14–C19) rings are inclined to the central 2*H*-1,2,3-triazole ring (N1–N3/C4/C5) by 1.80 (7) and 1.79 (7)°, respectively, and to one another by 2.16 (7)°. The nitro group is co-planar with the benzene ring (C14–C19) to which it is connected [torsion angles O1—N7—C17—C16 = 0.4 (2)° and O2—N7—C17—C16 = −179.18 (12)°]. The azido group (–N3=N4^+^=N5^−^) is almost co-planar with the central 2*H*-1,2,3-triazole ring to which it is connected [N6—N5—N4 = 171.56 (14)° and torsion angles N5—N4—C4—N3 = −1.69 (19)° and N5—N4—C4—C5 = 177.54 (13)°].

## 3. Supra­molecular features and Hirshfeld surface analysis

**Figure 3:**
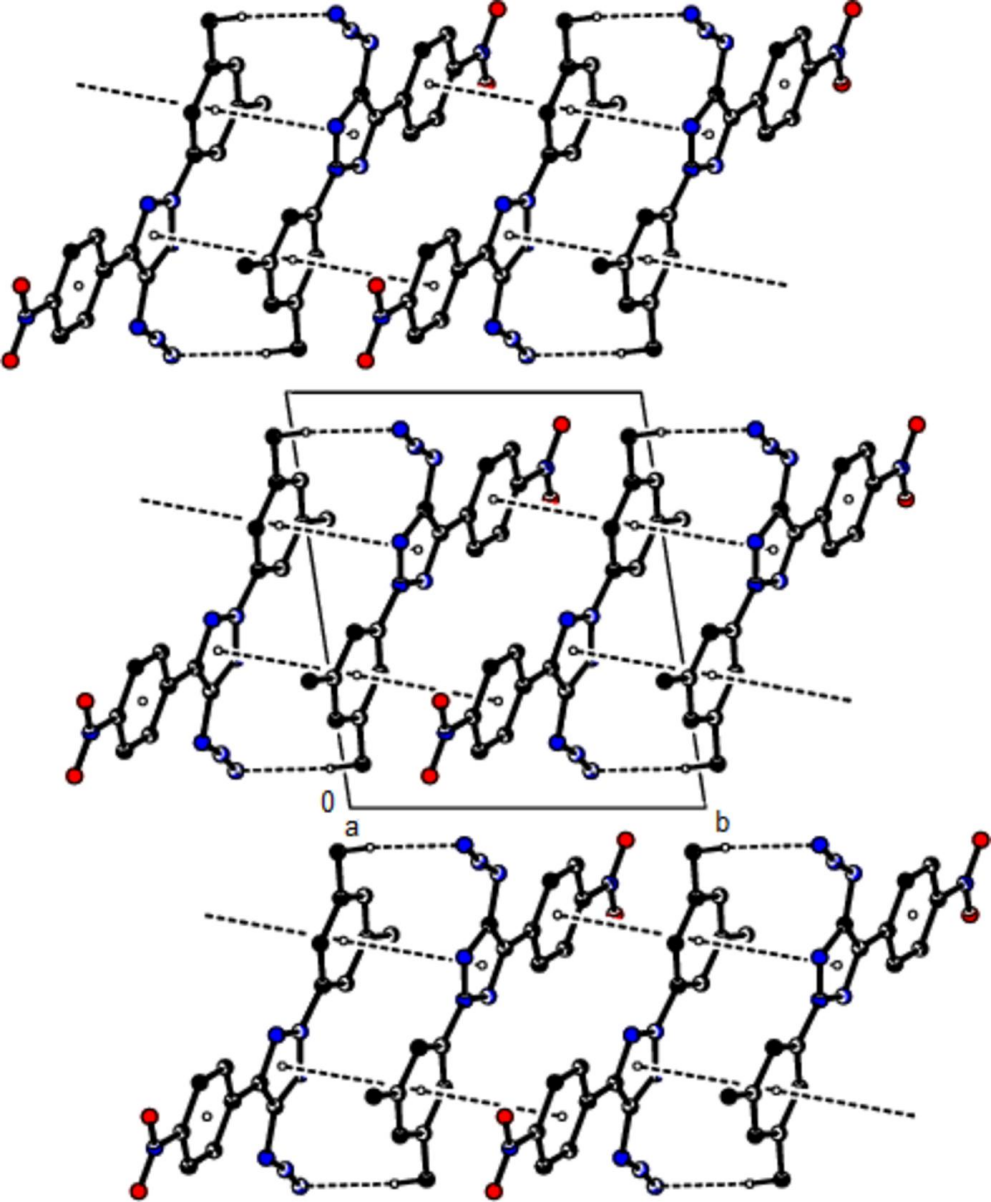
The crystal packing of the title compound viewed along the *a*-axis with inter­molecular C—H⋯N contacts and π–π stacking inter­actions shown as dashed lines.

In the crystal, the mol­ecules are linked by C—H⋯N hydrogen bonds and π–π stacking inter­actions [*Cg*1⋯*Cg*2^*i*^ = 3.7295 (9) Å; slippage = 1.489 Å and *Cg*2⋯*Cg*3^ii^ = 3.7971 (9) Å; slippage = 1.783 Å; symmetry codes: (i) −*x* + 1, −*y*, −*z* + 1, (ii) −*x* + 1, −*y* + 1, −*z* + 1; *Cg*1, *Cg*2 and *Cg*3 are the centroids of the 2*H*-1,2,3-triazole (N1–N3/C4/C5), 3,5-di­methyl­phenyl (C6–C11) and 4-nitro­phenyl (C14–C19) rings, respectively], forming ribbons along the *b*-axis direction (Tables 1 ▸ and 2 ▸; Fig. 3 ▸). These ribbons are connected to each other by weak van der Waals inter­actions and the stability of the crystal structure is ensured.

**Figure 4:**
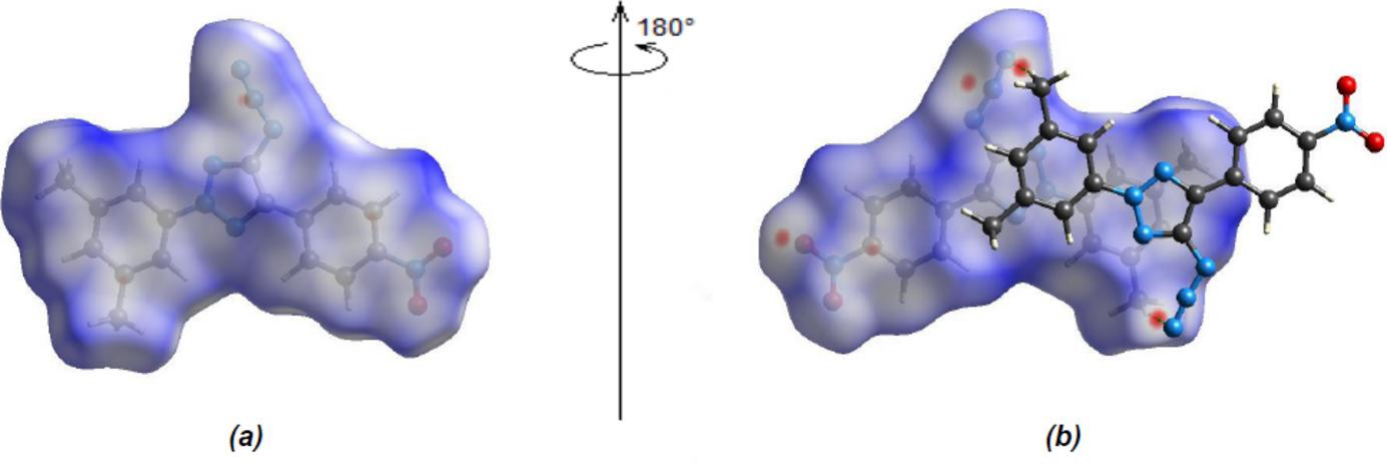
(*a*) Front and (*b*) back views of the three-dimensional Hirshfeld surface of the title compound plotted over *d*_norm_.

**Figure 5:**
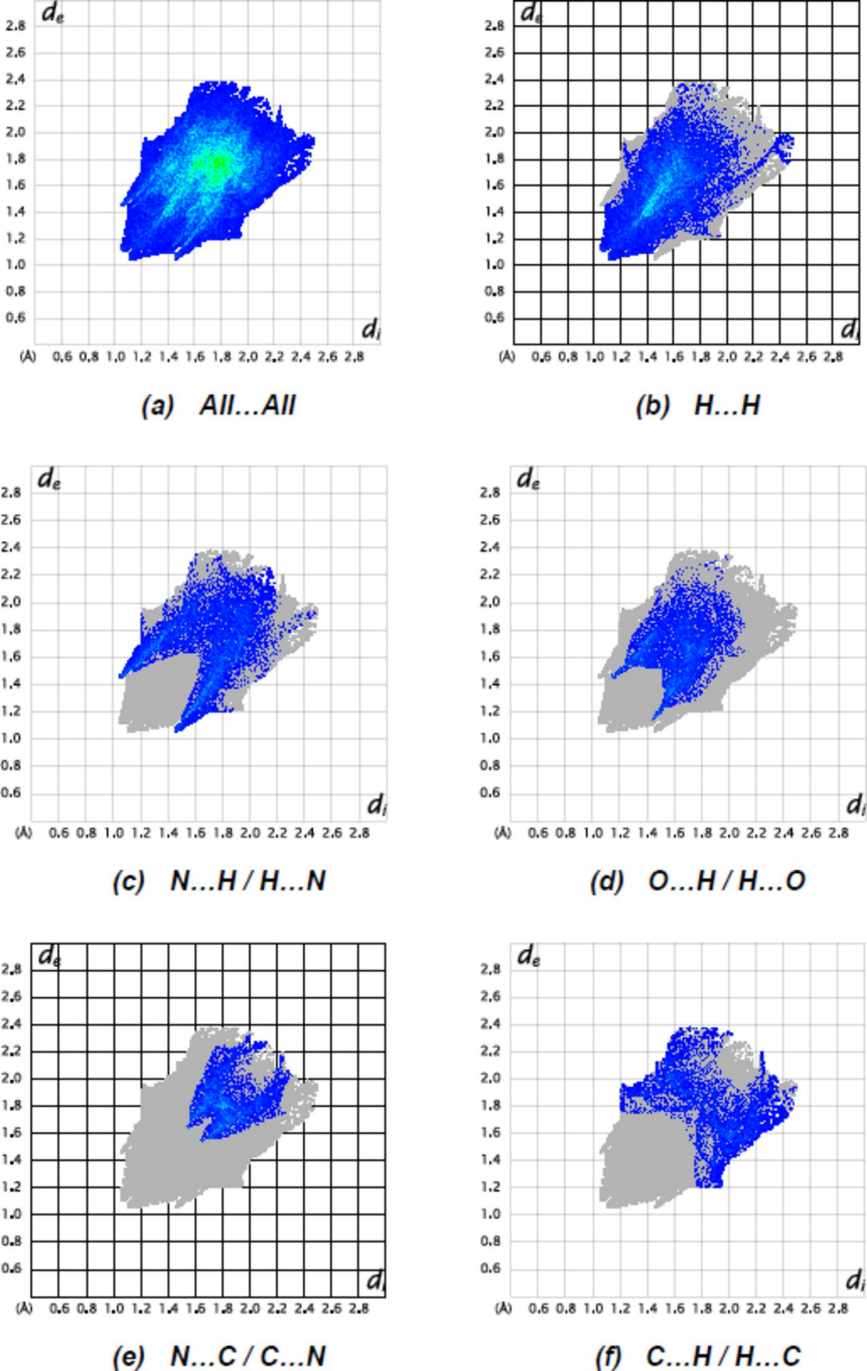
The full two-dimensional fingerprint plots for the title compound, showing (*a*) all inter­actions, and delineated into (*b*) H⋯H, (*c*) N⋯H/H⋯N, (*d*) O⋯H/H⋯O, (*e*) N⋯C/C⋯N and (*e*) C⋯H/H⋯C inter­actions. The *d*_i_ and *d*_e_ values are the closest inter­nal and external distances (in Å) from given points on the Hirshfeld surface.

In order to investigate the inter­molecular inter­actions in a visual manner, a Hirshfeld surface analysis was performed using *CrystalExplorer 17.5* (Spackman *et al.*, 2021 ▸). The bright-red spots on the Hirshfeld surface mapped over *d*_norm_ (Fig. 4 ▸) indicate the presence of C—H⋯N inter­actions. The fingerprint plots (Fig. 5 ▸) are given for all contacts, and those delineated into H⋯H (31.5%), N⋯H/H⋯N (19.2%), O⋯H/H⋯O (14.5%), N⋯C/C⋯N (10.9%), C⋯H/H⋯C (10.2%), C⋯C (5.2%), O⋯N/N⋯O (4.0%), O⋯C/C⋯O (2.4%) and N⋯N (2.1%). The most important contributions to the crystal packing are H⋯H and N⋯H/H⋯N contacts.

## 4. Database survey

The ten most similar compounds found in a search of the Cambridge Structural Database (CSD, Version 5.42, update of September 2021; Groom *et al.*, 2016 ▸) for the 2*H*-1,2,3-triazole group are JADSEP (Canseco-González *et al.*, 2015 ▸), JELTEC (Zukerman-Schpector *et al.*, 2017 ▸), HUYTEC (Haslinger *et al.*, 2015 ▸), FEVLIE, FEVLOK, FEVLUQ, FEVMAX, FEVMEB and FEVMOL (Farrán *et al.*, 2018 ▸) and SECQUO (Altimari *et al.*, 2012 ▸).

In the crystal of JADSEP, mol­ecules are linked *via* C—H⋯I hydrogen bonds, forming slabs parallel to the *ab* plane. Within the slabs there are weak π–π inter­actions present involving the mesityl and phenyl rings. In the crystal of JELTEC, the three-dimensional packing is stabilized by a combination of methyl­ene-C—H⋯O, methyl­ene-C—H⋯π, C—H⋯π and nitro-O⋯π (nitro­benzene) inter­actions, along with weak π (triazol­yl)–π (nitro­benzene) contacts. In the crystal of HUYTEC, the water mol­ecules are connected into [010] chains by O—H⋯O hydrogen bonds, while O—H⋯N hydrogen bonds connect the water mol­ecules to the organic mol­ecules, generating corrugated (100) sheets. In the crystals of FEVLIE, FEVLOK, FEVLUQ, FEVMAX, FEVMEB and FEVMOL, there are no C_ar_—H⋯F—C intra­molecular contacts. If the aryl groups were coplanar with the triazole ring, the C—F and the C—H atoms would be too close. Thus, the steric effect is more efficient than the weak hydrogen bond. Only compound FEVMOL clearly shows a hydrogen bond (O—H⋯N). In the crystal of SECQUO, the mol­ecules pack in a head-to-tail arrangement along the *a*-axis direction with closest inter-centroid distances between the triazole rings of 3.7372 (12) Å.

## 5. Synthesis and crystallization

The title compound was synthesized according to a literature protocol (Tsyrenova *et al.*, 2021 ▸). A 20 ml screw-neck vial was charged with DMSO (20 ml), (*E*)-1-[2,2-di­chloro-1-(4-nitro­phen­yl)vin­yl]-2-(3,5-di­methyl­phen­yl)diazene (350 mg, 1 mmol) and sodium azide (NaN_3_; 390 mq; 3 mmol). After 1–3 h (until TLC analysis showed complete consumption of the corresponding triazole), the reaction mixture was poured into a 0.01 *M* solution of HCl (100 ml, pH = 2–3), and extracted with di­chloro­methane (3 × 20 ml). The combined organic phase was washed with water (3 × 50 ml), brine (30 ml), dried over anhydrous Na_2_SO_4_ and concentrated *in vacuo* using a rotary evaporator. The residue was purified by column chromatography on silica gel using appropriate mixtures of hexane and di­chloro­methane (*v*/*v*: 3/1–1/1). Red solid (yield 75%); m.p. 375 K. Analysis calculated for C_16_H_13_N_7_O_2_ (*M* = 335.33): ^1^H NMR (300 MHz, chloro­form-*d*) δ 8.84 (*s*, 1H), 8.40–8.27 (*m*, 1H), 8.20 (*d*, *J* = 8.2 Hz, 1H), 7.68 (*s*, 2H), 7.62 (*t*, *J* = 8.0 Hz, 1H), 7.02 (*s*, 1H), 2.44 (*s*, 6H). ^13^C NMR (75 MHz, CDCl_3_) δ 143.9, 134.7, 134.4, 130.1, 127.3, 126.1, 125.0, 118.3, 116.5, 111.4, 16.8. The compound was dissolved in di­chloro­methane and then left at room temperature for slow evaporation; red crystal of the title compound suitable for X-rays started to form after *ca* 2 d.

## 6. Refinement

Crystal data, data collection and structure refinement details are summarized in Table 3 ▸. All H atoms were positioned geometrically and allowed to ride on their parent atoms (C—H = 0.95–0.98 Å) with *U*_iso_(H) = 1.2 or 1.5*U*_eq_(C). Owing to poor agreement between observed and calculated intensities, sixteen outliers (6 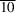 1, 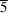 12 0, 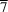 11 0, 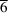 12 1, 6 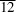 1, 7 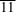 1, 5 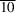 1, 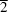 2 6, 2 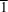 1, 1 1 4, 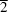 4 1, 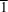 1 1, 0 2 0, 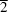 1 2, 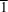 4 4, 2 0 5) were omitted during the final refinement cycle.

## Supplementary Material

Crystal structure: contains datablock(s) I. DOI: 10.1107/S2056989023007855/zn2031sup1.cif

Structure factors: contains datablock(s) I. DOI: 10.1107/S2056989023007855/zn2031Isup2.hkl

CCDC reference: 2293836

Additional supporting information:  crystallographic information; 3D view; checkCIF report

## Figures and Tables

**Table 1:** Hydrogen-bond geometry (Å, °)

*D*—H⋯*A*	*D*—H	H⋯*A*	*D*⋯*A*	*D*—H⋯*A*
C12—H12*A*⋯N6^i^	0.98	2.61	3.570 (2)	166

Symmetry code: (i) 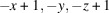 .

**Table 2:** Summary of short inter­atomic contacts (Å) in the title compound

O1⋯C12	3.36	*x*, 1 + *y*, 1 + *z*
N5⋯O1	2.98	1 − *x*, 1 − *y*, 2 − *z*
H13*A*⋯O2	2.90	1 − *x*, 1 − *y*, 1 − *z*
H7⋯H13*A*	2.59	−1 + *x*, *y*, *z*
H19⋯H18	2.37	−*x*, 1 − *y*, 1 − *z*
H12*A*⋯N6	2.61	1 − *x*, −*y*, 1 − *z*
H13*C*⋯H11	2.51	2 − *x*, −*y*, 1 − *z*
H15⋯N6	2.75	2 − *x*, 1 − *y*, 2 − *z*
H12*A*⋯C12	2.93	−*x*, −*y*, −*z*

**Table 3:** Experimental details

Crystal data	
Chemical formula	C_16_H_13_N_7_O_2_
*M*_r_	335.33
Crystal system, space group	Triclinic, *P* 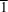
Temperature (K)	100
*a*, *b*, *c* (Å)	7.4400 (8), 9.920 (1), 11.5200 (13)
α, β, γ (°)	93.071 (9), 105.349 (10), 108.879 (11)
*V* (Å^3^)	766.71 (16)
*Z*	2
Radiation type	Synchrotron, λ = 0.79313 Å
μ (mm^−1^)	0.13
Crystal size (mm)	0.09 × 0.05 × 0.03

Data collection	
Diffractometer	Rayonix SX165 CCD
Absorption correction	Multi-scan (*SCALA*; Evans, 2006 ▸)
*T*_min_, *T*_max_	0.969, 0.990
No. of measured, independent and observed [*I* > 2σ(*I*)] reflections	13293, 3466, 2976
*R*_int_	0.025
(sin θ/λ)_max_ (Å^−1^)	0.650

Refinement	
*R*[*F* ^2^ > 2σ(*F* ^2^)], *wR*(*F* ^2^), *S*	0.046, 0.133, 1.08
No. of reflections	3466
No. of parameters	229
H-atom treatment	H-atom parameters constrained
Δρ_max_, Δρ_min_ (e Å^−3^)	0.27, −0.22

Computer programs: *Marccd* (Doyle, 2011 ▸), *iMosflm* (Battye *et al.*, 2011 ▸), *SHELXT* (Sheldrick, 2015*a* ▸), *SHELXL* (Sheldrick, 2015*b* ▸), *ORTEP-3 for Windows* (Farrugia, 2012 ▸) and *PLATON* (Spek, 2020 ▸).

## supplementary crystallographic information

### Crystal data

**Table:**

C_16_H_13_N_7_O_2_	*Z* = 2
*M**_r_* = 335.33	*F*(000) = 348
Triclinic, *P*1	*D*_x_ = 1.452 Mg m^−^^3^
*a* = 7.4400 (8) Å	Synchrotron radiation, λ = 0.79313 Å
*b* = 9.920 (1) Å	Cell parameters from 1000 reflections
*c* = 11.5200 (13) Å	θ = 2.1–28.0°
α = 93.071 (9)°	µ = 0.13 mm^−^^1^
β = 105.349 (10)°	*T* = 100 K
γ = 108.879 (11)°	Prism, red
*V* = 766.71 (16) Å^3^	0.09 × 0.05 × 0.03 mm

### Data collection

**Table:**

Rayonix SX165 CCD diffractometer	2976 reflections with *I* > 2σ(*I*)
/f scan	*R*_int_ = 0.025
Absorption correction: multi-scan (*SCALA*; Evans, 2006)	θ_max_ = 31.0°, θ_min_ = 2.1°
*T*_min_ = 0.969, *T*_max_ = 0.990	*h* = −9→9
13293 measured reflections	*k* = −12→12
3466 independent reflections	*l* = −14→14

### Refinement

**Table:**

Refinement on *F*^2^	Secondary atom site location: difference Fourier map
Least-squares matrix: full	Hydrogen site location: inferred from neighbouring sites
*R*[*F*^2^ > 2σ(*F*^2^)] = 0.046	H-atom parameters constrained
*wR*(*F*^2^) = 0.133	*w* = 1/[σ^2^(*F*_o_^2^) + (0.0764*P*)^2^ + 0.1976*P*] where *P* = (*F*_o_^2^ + 2*F*_c_^2^)/3
*S* = 1.08	(Δ/σ)_max_ < 0.001
3466 reflections	Δρ_max_ = 0.27 e Å^−^^3^
229 parameters	Δρ_min_ = −0.22 e Å^−^^3^
0 restraints	Extinction correction: SHELXL, Fc^*^=kFc[1+0.001xFc^2^λ^3^/sin(2θ)]^-1/4^
Primary atom site location: difference Fourier map	Extinction coefficient: 0.074 (7)

### Special details

**Table:**

Geometry. All esds (except the esd in the dihedral angle between two l.s. planes) are estimated using the full covariance matrix. The cell esds are taken into account individually in the estimation of esds in distances, angles and torsion angles; correlations between esds in cell parameters are only used when they are defined by crystal symmetry. An approximate (isotropic) treatment of cell esds is used for estimating esds involving l.s. planes.

### Fractional atomic coordinates and isotropic or equivalent isotropic displacement parameters (Å^2^)

**Table:**

	*x*	*y*	*z*	*U*_iso_*/*U*_eq_	
O1	0.18207 (18)	0.76197 (12)	0.92289 (10)	0.0446 (3)	
O2	−0.03369 (17)	0.69879 (12)	0.74319 (11)	0.0423 (3)	
N1	0.47322 (16)	0.30620 (12)	0.54670 (10)	0.0295 (3)	
N2	0.59776 (16)	0.23870 (12)	0.53829 (10)	0.0280 (3)	
N3	0.74669 (16)	0.25675 (12)	0.64053 (10)	0.0295 (3)	
N4	0.83239 (17)	0.38805 (13)	0.84246 (10)	0.0326 (3)	
N5	0.96905 (17)	0.34031 (13)	0.87091 (10)	0.0332 (3)	
N6	1.10029 (19)	0.30585 (14)	0.91074 (12)	0.0387 (3)	
N7	0.11839 (19)	0.69804 (13)	0.81792 (11)	0.0343 (3)	
C4	0.71164 (19)	0.34137 (14)	0.71900 (12)	0.0291 (3)	
C5	0.54163 (19)	0.37371 (14)	0.66201 (12)	0.0282 (3)	
C6	0.57495 (19)	0.15443 (14)	0.42750 (12)	0.0279 (3)	
C7	0.41649 (19)	0.14311 (14)	0.32646 (12)	0.0284 (3)	
H7	0.3240	0.1885	0.3323	0.034*	
C8	0.3946 (2)	0.06480 (14)	0.21667 (12)	0.0305 (3)	
C9	0.5333 (2)	−0.00175 (14)	0.21177 (13)	0.0325 (3)	
H9	0.5185	−0.0562	0.1373	0.039*	
C10	0.6915 (2)	0.00999 (15)	0.31301 (13)	0.0331 (3)	
C11	0.7126 (2)	0.08921 (15)	0.42256 (13)	0.0323 (3)	
H11	0.8196	0.0984	0.4929	0.039*	
C12	0.2269 (2)	0.05374 (16)	0.10597 (13)	0.0361 (3)	
H12A	0.1186	−0.0385	0.0952	0.054*	
H12B	0.2744	0.0595	0.0341	0.054*	
H12C	0.1783	0.1330	0.1163	0.054*	
C13	0.8416 (2)	−0.05959 (18)	0.30537 (15)	0.0420 (4)	
H13A	0.9732	0.0151	0.3219	0.063*	
H13B	0.8026	−0.1137	0.2236	0.063*	
H13C	0.8465	−0.1254	0.3657	0.063*	
C14	0.43732 (19)	0.45871 (14)	0.70518 (12)	0.0280 (3)	
C15	0.5043 (2)	0.53230 (15)	0.82427 (12)	0.0319 (3)	
H15	0.6221	0.5286	0.8796	0.038*	
C16	0.4004 (2)	0.61052 (15)	0.86228 (12)	0.0330 (3)	
H16	0.4453	0.6600	0.9433	0.040*	
C17	0.2301 (2)	0.61509 (14)	0.77992 (12)	0.0303 (3)	
C18	0.1596 (2)	0.54308 (15)	0.66123 (13)	0.0326 (3)	
H18	0.0421	0.5477	0.6063	0.039*	
C19	0.2631 (2)	0.46465 (15)	0.62443 (12)	0.0317 (3)	
H19	0.2160	0.4142	0.5437	0.038*	

### Atomic displacement parameters (Å^2^)

**Table:**

	*U*^11^	*U*^22^	*U*^33^	*U*^12^	*U*^13^	*U*^23^
O1	0.0571 (7)	0.0466 (6)	0.0388 (6)	0.0239 (5)	0.0224 (5)	0.0007 (5)
O2	0.0407 (6)	0.0442 (6)	0.0509 (6)	0.0247 (5)	0.0162 (5)	0.0061 (5)
N1	0.0288 (5)	0.0328 (6)	0.0295 (6)	0.0152 (5)	0.0077 (4)	0.0019 (4)
N2	0.0263 (5)	0.0323 (6)	0.0273 (5)	0.0144 (4)	0.0065 (4)	0.0030 (4)
N3	0.0273 (5)	0.0334 (6)	0.0283 (6)	0.0133 (4)	0.0059 (4)	0.0041 (4)
N4	0.0292 (6)	0.0376 (6)	0.0305 (6)	0.0154 (5)	0.0050 (5)	−0.0014 (5)
N5	0.0331 (6)	0.0367 (6)	0.0288 (6)	0.0134 (5)	0.0065 (5)	0.0032 (5)
N6	0.0351 (6)	0.0455 (7)	0.0364 (6)	0.0196 (5)	0.0057 (5)	0.0058 (5)
N7	0.0402 (6)	0.0317 (6)	0.0383 (6)	0.0160 (5)	0.0193 (5)	0.0052 (5)
C4	0.0276 (6)	0.0317 (6)	0.0286 (6)	0.0123 (5)	0.0069 (5)	0.0046 (5)
C5	0.0278 (6)	0.0304 (6)	0.0272 (6)	0.0116 (5)	0.0077 (5)	0.0035 (5)
C6	0.0290 (6)	0.0288 (6)	0.0290 (6)	0.0127 (5)	0.0106 (5)	0.0035 (5)
C7	0.0268 (6)	0.0289 (6)	0.0311 (7)	0.0117 (5)	0.0091 (5)	0.0027 (5)
C8	0.0298 (6)	0.0294 (6)	0.0320 (7)	0.0104 (5)	0.0093 (5)	0.0026 (5)
C9	0.0360 (7)	0.0306 (6)	0.0344 (7)	0.0139 (5)	0.0143 (6)	0.0014 (5)
C10	0.0342 (7)	0.0332 (7)	0.0379 (7)	0.0167 (6)	0.0146 (6)	0.0052 (5)
C11	0.0306 (6)	0.0369 (7)	0.0334 (7)	0.0174 (6)	0.0093 (5)	0.0058 (5)
C12	0.0359 (7)	0.0384 (7)	0.0314 (7)	0.0152 (6)	0.0049 (6)	−0.0029 (5)
C13	0.0411 (8)	0.0484 (8)	0.0449 (8)	0.0268 (7)	0.0136 (6)	0.0019 (7)
C14	0.0279 (6)	0.0292 (6)	0.0273 (6)	0.0110 (5)	0.0080 (5)	0.0031 (5)
C15	0.0312 (6)	0.0357 (7)	0.0279 (6)	0.0133 (5)	0.0056 (5)	0.0021 (5)
C16	0.0366 (7)	0.0344 (7)	0.0278 (6)	0.0131 (6)	0.0091 (5)	0.0004 (5)
C17	0.0329 (7)	0.0301 (6)	0.0324 (7)	0.0136 (5)	0.0139 (5)	0.0038 (5)
C18	0.0310 (6)	0.0366 (7)	0.0320 (7)	0.0160 (5)	0.0075 (5)	0.0028 (5)
C19	0.0319 (7)	0.0361 (7)	0.0275 (6)	0.0159 (6)	0.0055 (5)	−0.0002 (5)

### Geometric parameters (Å, º)

**Table:**

O1—N7	1.2284 (16)	C9—H9	0.9500
O2—N7	1.2296 (17)	C10—C11	1.3944 (19)
N1—N2	1.3261 (15)	C10—C13	1.5085 (19)
N1—C5	1.3410 (16)	C11—H11	0.9500
N2—N3	1.3435 (15)	C12—H12A	0.9800
N2—C6	1.4268 (17)	C12—H12B	0.9800
N3—C4	1.3323 (17)	C12—H12C	0.9800
N4—N5	1.2317 (16)	C13—H13A	0.9800
N4—C4	1.4242 (17)	C13—H13B	0.9800
N5—N6	1.1299 (17)	C13—H13C	0.9800
N7—C17	1.4681 (17)	C14—C15	1.3996 (18)
C4—C5	1.4069 (18)	C14—C19	1.4020 (18)
C5—C14	1.4640 (18)	C15—C16	1.3866 (19)
C6—C11	1.3876 (18)	C15—H15	0.9500
C6—C7	1.3897 (18)	C16—C17	1.383 (2)
C7—C8	1.3905 (18)	C16—H16	0.9500
C7—H7	0.9500	C17—C18	1.3886 (19)
C8—C9	1.4043 (19)	C18—C19	1.3798 (19)
C8—C12	1.4995 (19)	C18—H18	0.9500
C9—C10	1.389 (2)	C19—H19	0.9500
			
N2—N1—C5	104.92 (11)	C6—C11—H11	120.4
N1—N2—N3	115.31 (11)	C10—C11—H11	120.4
N1—N2—C6	121.86 (11)	C8—C12—H12A	109.5
N3—N2—C6	122.82 (11)	C8—C12—H12B	109.5
C4—N3—N2	102.74 (11)	H12A—C12—H12B	109.5
N5—N4—C4	113.98 (11)	C8—C12—H12C	109.5
N6—N5—N4	171.56 (14)	H12A—C12—H12C	109.5
O1—N7—O2	123.90 (12)	H12B—C12—H12C	109.5
O1—N7—C17	117.93 (12)	C10—C13—H13A	109.5
O2—N7—C17	118.17 (12)	C10—C13—H13B	109.5
N3—C4—C5	110.21 (11)	H13A—C13—H13B	109.5
N3—C4—N4	122.95 (12)	C10—C13—H13C	109.5
C5—C4—N4	126.83 (12)	H13A—C13—H13C	109.5
N1—C5—C4	106.82 (11)	H13B—C13—H13C	109.5
N1—C5—C14	120.20 (12)	C15—C14—C19	119.20 (12)
C4—C5—C14	132.97 (12)	C15—C14—C5	122.24 (12)
C11—C6—C7	121.96 (12)	C19—C14—C5	118.56 (12)
C11—C6—N2	119.59 (12)	C16—C15—C14	120.60 (13)
C7—C6—N2	118.43 (11)	C16—C15—H15	119.7
C6—C7—C8	119.39 (12)	C14—C15—H15	119.7
C6—C7—H7	120.3	C17—C16—C15	118.67 (12)
C8—C7—H7	120.3	C17—C16—H16	120.7
C7—C8—C9	118.67 (13)	C15—C16—H16	120.7
C7—C8—C12	120.20 (12)	C16—C17—C18	122.11 (13)
C9—C8—C12	121.13 (12)	C16—C17—N7	119.61 (12)
C10—C9—C8	121.71 (13)	C18—C17—N7	118.28 (12)
C10—C9—H9	119.1	C19—C18—C17	118.84 (13)
C8—C9—H9	119.1	C19—C18—H18	120.6
C9—C10—C11	119.17 (13)	C17—C18—H18	120.6
C9—C10—C13	120.99 (13)	C18—C19—C14	120.58 (12)
C11—C10—C13	119.84 (13)	C18—C19—H19	119.7
C6—C11—C10	119.10 (13)	C14—C19—H19	119.7
			
C5—N1—N2—N3	−0.26 (15)	C8—C9—C10—C11	0.4 (2)
C5—N1—N2—C6	−179.58 (11)	C8—C9—C10—C13	−178.68 (13)
N1—N2—N3—C4	0.38 (15)	C7—C6—C11—C10	0.0 (2)
C6—N2—N3—C4	179.70 (12)	N2—C6—C11—C10	−178.46 (12)
N2—N3—C4—C5	−0.34 (14)	C9—C10—C11—C6	−0.1 (2)
N2—N3—C4—N4	179.00 (12)	C13—C10—C11—C6	178.98 (13)
N5—N4—C4—N3	−1.69 (19)	N1—C5—C14—C15	−179.28 (12)
N5—N4—C4—C5	177.54 (13)	C4—C5—C14—C15	1.9 (2)
N2—N1—C5—C4	0.03 (14)	N1—C5—C14—C19	1.52 (19)
N2—N1—C5—C14	−179.08 (11)	C4—C5—C14—C19	−177.31 (14)
N3—C4—C5—N1	0.21 (15)	C19—C14—C15—C16	−0.1 (2)
N4—C4—C5—N1	−179.10 (12)	C5—C14—C15—C16	−179.32 (12)
N3—C4—C5—C14	179.16 (13)	C14—C15—C16—C17	−0.4 (2)
N4—C4—C5—C14	−0.1 (2)	C15—C16—C17—C18	0.5 (2)
N1—N2—C6—C11	178.22 (12)	C15—C16—C17—N7	−179.59 (12)
N3—N2—C6—C11	−1.1 (2)	O1—N7—C17—C16	0.4 (2)
N1—N2—C6—C7	−0.33 (19)	O2—N7—C17—C16	−179.18 (12)
N3—N2—C6—C7	−179.60 (11)	O1—N7—C17—C18	−179.69 (12)
C11—C6—C7—C8	−0.3 (2)	O2—N7—C17—C18	0.70 (19)
N2—C6—C7—C8	178.25 (12)	C16—C17—C18—C19	−0.1 (2)
C6—C7—C8—C9	0.5 (2)	N7—C17—C18—C19	−179.95 (12)
C6—C7—C8—C12	−178.79 (12)	C17—C18—C19—C14	−0.5 (2)
C7—C8—C9—C10	−0.6 (2)	C15—C14—C19—C18	0.6 (2)
C12—C8—C9—C10	178.72 (13)	C5—C14—C19—C18	179.82 (12)

### Hydrogen-bond geometry (Å, º)

**Table:**

*D*—H···*A*	*D*—H	H···*A*	*D*···*A*	*D*—H···*A*
C7—H7···N1	0.95	2.47	2.7900 (18)	100
C12—H12*A*···N6^i^	0.98	2.61	3.570 (2)	166
C15—H15···N4	0.95	2.51	3.174 (2)	127
C19—H19···N1	0.95	2.47	2.810 (2)	101

Symmetry code: (i) −*x*+1, −*y*, −*z*+1.
